# A Controversial Relationship Between Crohn’s Disease and Hidradenitis Suppurativa: A Case Series and Literature Review

**DOI:** 10.7759/cureus.23422

**Published:** 2022-03-23

**Authors:** Faith Buchanan, Saad Saleem, Mohd Amer Alsamman

**Affiliations:** 1 Internal Medicine, MedStar Washington Hospital Center, Washington, D.C., USA; 2 Internal Medicine, Sunrise Hospital and Medical Center, Las Vegas, USA; 3 Gastroenterology and Hepatology, MedStar Georgetown University Hospital, Washington, D.C., USA

**Keywords:** hidradenitis suppurative (hs), inflammatory bowel disease, anti-tnf alpha, biological therapy, crohn’s disease (cd)

## Abstract

Crohn's disease (CD) is an inflammatory bowel disease (IBD) with major extraintestinal manifestations. Hidradenitis suppurativa (HS) is a chronic inflammatory skin condition that has previously been described to have a strong association with CD. Though the pathophysiology remains uncertain, this case series highlights the different aspects of disease presentation, similarities, severity, current treatment modalities, and the relational conflict between HS as a paradoxical side effect of biologic agents (BA) that is not well established.

We identified a total of three patients with CD and HS and described their clinical presentation and management. A systematic search of the literature with PubMed and Ovid MEDLINE was done in 2021. Two patients were initially diagnosed with CD prior to developing skin manifestations. The third patient was diagnosed with HS first, then was found to have gastrointestinal symptoms. All patients had HS requiring surgical intervention. One patient failed a biological agent but responded to another. The second patient was treated with cytotoxic agents with acceptable results. The third patient was managed without the use of biologics. One of three patients’ clinical courses may suggest a paradoxical side effect of BA.

The relationship between CD and HS is based on several case reports. A prospective study will help establish the relationship as well as shed light on the treatment of both conditions simultaneously. In addition, further evaluation of the causal relationship between BA, specifically adalimumab and infliximab as treatment for CD and HS are warranted to effectively manage Crohn’s disease, evaluate paradoxical HS, and improve outcomes of both HS and CD. CD and HS impact a patient's quality of life and physicians should therefore have a high degree of awareness upon diagnosis.

## Introduction

Crohn’s disease (CD) is an inflammatory bowel disease (IBD) of uncertain etiology characterized by transmural inflammation that may involve the entire gastrointestinal tract from the mouth to the perianal area. Approximately 80% of patients have small bowel involvement, usually in the distal ileum, with one-third of patients having ileitis exclusively. Major extraintestinal manifestations of CD include joint, eye, and skin [1,2]. Hidradenitis suppurativa (HS) is a chronic, relapsing, and painful inflammatory skin condition. Primary sites of involvement are skin folds with terminal hair follicles such as the axilla, the genitoinguinal, and anogenital regions [3]. Previous studies have approximated the prevalence of HS as 1-4% with a higher female preponderance, and the disease developing predominantly in the early 20s [4]. The clinical manifestations can greatly vary from patients having recurrent inflamed nodules and abscesses to draining sinus tracts and bands of severe scar formation. In addition, tumor necrosis factor (TNF) has been demonstrated to have a role in the pathogenesis of HS lesions, with circulating levels suggestive of active inflammation [1,5].

Although CD and HS are distinctive diseases, both share several features suggestive of common underlying pathophysiology. For example, risk factors for both HS and CD include diabetes, female gender, obesity, and smoking. Tumor necrosis factors (TNF) inhibitors have also been used to treat CD and improve HS that is resistant to other treatment modalities [2,3,6]. Recent studies have suggested a paradoxical side effect of HS in patients with CD on biologic agents (BA), specifically adalimumab and infliximab [7,8]. However, this relationship is not well established.

## Case presentation

Case 1 

A 23-year-old Caucasian female smoker had a history of long-standing small bowel fistulizing Crohn’s disease complicated by recurrent flares despite being treated with mesalamine, methotrexate (MTX), 6-mercaptopurine (6MP), and adalimumab. Several years following her diagnosis, she developed axillary and perineal severe nodules histologically consistent with hidradenitis suppurativa. She received treatment with antibiotics and surgery with only partial relief of symptoms. Recurrence of HS and CD flares continued to occur. She was started on certolizumab given at weeks zero, two, and four, then every 30 days as maintenance. Three years have passed after starting certolizumab with symptoms of both CD and HS being well controlled. 

Case 2 

A 37-year-old Caucasian female with a history of tobacco use and CD with perianal involvement was treated with mesalamine and MTX. Five years following diagnosis, she developed groin and perianal cutaneous lesions with purulent fluctuant painful nodules. The histological picture after biopsy confirmed HS with no granuloma. Several topical and systemic antibiotic treatments were tried with moderate response. Her lesions required surgical intervention with skin grafting. After achieving a partial remission of HS, she had a flare of CD that was managed with intravenous steroids, and eventually, infliximab was initiated with control of both conditions. 

Case 3 

A 28-year-old African American male with no prior smoking history presented with severe HS. He was first treated with topical and systemic antibiotics but had progression to involve the axillary, groin, and back of his neck requiring surgical excision and skin grafting. During his hospitalization, he was found to have iron deficiency anemia with a hemoglobin value level of 9 g/dL. Further history was notable for episodes of diarrhea, abdominal cramps, and blood in his stools. After discharge, the patient was referred to gastroenterology. A colonoscopy revealed mucosal edema and inflammation with skip ulcerations suggestive of Crohn’s disease throughout the colon. Pathology reports showed focal ulceration with evidence of chronic inflammation; a diagnosis of CD was made. 

## Discussion

Hidradenitis suppurativa (HS) and Crohn's disease are both chronic, progressive inflammatory conditions with an association first noticed in the 1990s [9]. This report describes three patients with underlying CD and HS, highlighting the shared characteristics often found, as well as recent concern for a paradoxical effect of biologic agents contributing to the development of HS in CD patients. Patients are more likely to be overweight/obese, to be former or current smokers, and to have ileocolonic and/or perianal disease. There have been several studies in the literature highlighting the association between HS and CD. Like our cases, patients may initially present with symptoms of either CD or HS. In a population-based study from the Mayo Clinic, the HS point prevalence among 679 patients with IBD was 1.2%, with the relative risk of developing HS with a diagnosis of CD nine times higher compared with the general population [10]. Again, a significant association with obesity, female sex, and perianal CD disease was found. Further, cross-sectional analysis has suggested that patients with underlying HS have three times the odds of having CD as those without HS [11].

Tobacco smoking also appears to be linked to both CD and HS as severity and incidence of disease tend to be higher among smokers compared to nonsmokers [11,12]. In a meta-analysis, smokers with Crohn's disease had a more complicated disease course and were at an increased risk of disease activity and flares. Similarly, smoking has been a well-documented risk factor for disease severity and development of HS [11]. According to Zhang et al., there have been several case reports indicating similar pathologic features between HS and CD. Furthermore, patients with both CD and HS symptoms experienced significant symptomatic relief with anti-TNF-a therapy, supporting the notion of common signaling pathways and immune-mediated origins of TNF-a and IL-23/Th17 [13].

As illustrated in the cases above, the management of HS usually consists of a stepwise approach with a combination of both medical therapies and surgical interventions. The major goal is to reduce inflammation-related pain and purulent discharge in hopes of improving a patient's quality of life and limiting the incidence and duration of flares. First-line treatment options include the use of local or systemic antibiotics with anti-inflammatory properties, such as tetracycline and the combination of clindamycin and rifampicin. More recently, biologics, such as anti-TNF-α agents (i.e., adalimumab and infliximab) which are common stay for patients with IBD, have also been shown to be effective in attaining relief in moderate-to-severe HS [14].

Adalimumab is the only US FDA and European Medicines Agency (EMA)-approved biologic for the treatment of HS, theoretically making adalimumab or BA a promising therapy for patients with HS and CD. In a randomized control trial performed by Kimball et al. in 2012, 154 adult patients with moderate-to-severe HS, unresponsive or intolerant to oral antibiotics were assigned to receive adalimumab 40 mg per week (adalimumab 40 mg every other week or placebo). Results showed that both adalimumab groups (17.6%, 9.6%, 3.9%) achieved better clinical response versus placebo, concluding that adalimumab dosed once weekly alleviates moderate-to-severe HS [15]. Based on recent literature review, there is a concern for a paradoxical side effect of biological agents, specifically adalimumab and infliximab, causing HS in patients with CD [7].

However, this proposed relationship is not well established and could merely be a failure of therapy in CD-treated patients, or simply highlighting the natural association of HS with treatment. In reviewing our cases, we see both cases 1 and 2 developed HS after established CD. Only in case 1, a patient with long-standing small bowel fistulizing CD and recurrent flares required treatment with adalimumab prior to the diagnosis of HS. This timeline may suggest a paradoxical effect of adalimumab. Conversely, in case 2, a patient with perianal CD treated initially with mesalamine and MTX was diagnosed with biopsy-confirmed HS prior to treatment with Infliximab is contradictory to HS as a paradoxical effect. Case 3 further supports an inadvertent relationship evident by diagnosis of HS prior to diagnosis of CD. It is unknown if case 1 and other cases of HS were solely in the setting of BA use. Overall, there is a relational conflict that exists as prevalence of diagnosis of HS in CD patients without the use of BA is higher versus prevalence of rare reported paradoxical cases.

Furthermore, HS and CD have similar pathology and common signaling pathways including TNF-a, with known effectiveness of anti-TNF-a agents as treatment of HS, therefore rebutting the notion of HS as a paradoxical effect of BA. Paradoxical HS induced by BA is scarcely reported and not well established. There have been several case reports and retrospective studies highlighting the association between HS and CD, thus it is possible that the development of HS after CD is a natural disease progression or cutaneous manifestation (Table 1).

**Table 1:** Literature review of CD and HD reported cases. *van der Zee et al. interviewed 102 CD patients about recurrent painful boils in the axillae and/or groin; the study showed that 17% of CD patients had a history compatible with HS, again suggesting an association between HS and CD [20]. **Church et al. performed a retrospective review of hospital records of 61 HS patients and found that 24 also had a diagnosis of CD. The diagnosis of CD predated that of HS by an average of 3.5 years [29]. CD: Crohn's disease; HS: hidradenitis suppurativa; F: female; M: male

Author	Year	Design	No.	A: HS then CD; B: CD then HS	Sex	Anti-TNF alpha refractory: (R)required second-line treatment
Ostlere et al. [9]	1991	Case report	3	B, B, B	F, F, M	N/A
Martinez et al. [14]	2001	Case report	1	B	F	Infliximab
Blazquez et al. [16]	2013	Case report	1	B	F	Infliximab
dos Santos et al. [17]	2012	Case report	1	B	F	Infliximab(R) - adalimumab
Koilakou et al. [18]	2010	Case report	1	B	M	Infliximab(R) - adalimumab
Yazdanyar et al. [19]	2010	Case report	2	A, A	F, F	Infliximab
van der Zee et al. [20]	2009	Retrospective	102	-*	M, F	N/A
Goertz et al. [21]	2009	Case report	1	B	M	Infliximab(R)
Rosi et al. [22]	2005	Case report	1	B	F	Infliximab
Roussomoustakaki et al. [23]	2003	Case report	1	A	F	Infliximab
Katsanos et al. [24]	2002	Case report	1	B	M	Infliximab
Roy et al. [25]	1997	Case report	1	A	M	N/A
Tsianos et al. [26]	1995	Case report	1	B	M	N/A
Kafity et al. [27]	1993	Case report	1	B	M	N/A
Attanoos et al. [28]	1993	Case report	3	B, A, B	F, M, M	N/A
Church et al. [29]	1993	Retrospective	61	-**	M, F	N/A
Cosnes et al. [30]	2011	Case report	3	B, B, B	M	N/A
Burrows et al. [31]	1992	Case report	2	A, A	M, F	N/A

CD and HS share several features including pathophysiology and risk factors, suggestive of disease relationship. However, this is primarily based on case reports. The causal relationship between BA-treated CD and HS is not well established nor is the paradoxical effect of BA with CD. A prospective study is warranted to establish the relation, optimal management of both conditions, and further evaluate the causal relationship between BA as a treatment for CD and paradoxical HS to improve physicians’ awareness and patients’ quality of life.

Key characteristics of three cases

Two Females and One Male

The prevalence of both conditions is higher in females. All three had CD and severe HS. The first two cases had a diagnosis of CD first. The third case had a diagnosis of HS first that led to investigation and diagnosis of CD. The first two cases were smokers. The third patient is morbidly obese which is a risk factor is for HS. All three did not have a family history of either HS or CD. Complicated management of HS required antibiotics (topical and systemic) and surgical intervention. Histological examination of excised skin lesions was diagnostic of HS with no evidence of granuloma. The first case received biological treatment with impressive improvement of both HS and CD. Initial control of CD symptoms achieved with adalimumab then diagnosis of severe resistant HS and relapse of CD. Certolizumab was used with stabilization of both conditions.

## Conclusions

Extra gastrointestinal manifestations occur frequently in inflammatory bowel disease. This case series highlights the association between CD and HS. Therefore, patients with HS and underlying gastrointestinal symptoms or signs of inflammatory bowel disease require additional evaluation by a gastroenterologist to prevent inappropriate or sub-optimal treatment as the coexistence of both disease processes can greatly impair a patient’s quality of life. Biological therapy is a common management strategy for both CD and HS. Adalimumab is the only FDA-approved BA for the management of HS and has demonstrated alleviation of moderate-to-severe HS. However, further evaluation of the causal relationship between BA, specifically adalimumab, infliximab, and HS is warranted to effectively manage Crohn’s disease and minimize cases of paradoxical HS while improving outcomes of both HS and CD.
